# Notes for Contributors

**DOI:** 10.1155/S1463924679000213

**Published:** 1979


continuous flow was introduced over 21 years ago. Many of the products shown at the associated exhibitions were exhibited for the first time in the UK. The fast-HPLC system, represents a considerable advance in the thinking of commercial companies. Automation in chromatography both gas and liquid has long been misconstrued as being simply mechanical sample injection coupled to electronic data acquisition and reduction. The system designed by Technicon attempts to cover the full analytical problem from sample handling and pretreatment through to measurement and data reduction. The solution is however, a complex instrument which by its nature is a combination of both automatic chemistry and chromatography. This highlights one of the perenial problems associated with automation in general; that is, who operates the instrument and to whom is it marketed? The operation of the instrument may well be within the compass of a junior analyst but the successful long term operation of the instrument will demand the presence of a more highly skilled scientist. This specialist must have an indepth understanding of the system, be able to detect and correct faulty operation and be able to undertake modifications and adjustments to meet new circumstances, such as significant changes in sample composition, and to meet unusual situations. In the fast-HPLC system the need is to both understand the implications of the sample pretreatment system and the chromatography involved. The instrument is an automatic system and not an adjunct to a chromatograph. Incorrect control of the first stages will negate the usefulness of the chromatographic measurements. In vitamin analysis for example it is very easy to produce incorrect results, if one or other of the vitamins being analysed is unstable to the chemical environment. However, these problems should not distract from the value of such degrees of automation since these are the correct way to overcome many of the bottlenecks in the laboratory. It is vital that the implications of their use are recognised and the proper degree of training provided. Regarding the system as a fast-HPLC system and not as an automatic instrument system does not seem to be the most satisfactory approach.

The aim of this journal is to improve the understanding of the philosophy of automation and from time to time focus on training and education problems. In this respect the UK Chemical Society is sponsoring a summer school on Automatic Analysis. This and Analysis 1979, sponsored by this journal, provide two additional vehicles to improve the communications in the area.

The journal will flourish best given a lively interaction with the readership. Hopefully the content of this and future issues will encourage you to write your views and comments to us. We look forward to a full postbag in 1979.

*Peter B. Stockwell*

| Name | Country | Work Experience | | | | | Discipline of Application | | | | |
|---|---|---|---|---|---|---|---|---|---|---|---|
| | | Academic | Research Institute | Government | Commercial | | Clinical Chem | Process Control | Industrial Application | Pharmaceutical | Clin/Indust |
| Stockwell | UK | | | × | | | | | × | | |
| Arndt | Switz | | | | × | | | | × | | |
| Malmstadt | USA | × | | | | | | | | | × |
| Mitchell | UK | | × | | | | × | | | | |
| Burtiss | USA | | × | | | | × | | | | |
| Fearn | UK | | | | × | | | | × | | |
| Greary | Aust | | × | | | | × | | | | |
| Haeckel | Germ | × | | | | | × | | | | |
| Kenyhertz | USA | | | | × | | | | × | | |
| Okuda | Japan | × | | | | | × | | | | |
| Nadean | USA | | | | × | | × | | | | |
| Pardue | USA | × | | | | | | | | | × |
| Porter | UK | | | × | | | | | × | | |
| Roberts | UK | | × | | | | × | | | | |
| Simon | Switz | × | | | | | | | × | | |
| Squirell | UK | | | | × | | | | × | | |
| Stewart | USA | | | × | | | | | × | | |
| Werder | Switz | | | | × | | | | × | | |

### Notes for Contributors

### Presentation of manuscripts

Manuscripts should be typed (double-spaced) on one side of the paper only and with generous margins. The title should be brief and informative avoiding the word "new" and its synonyms. The full list of authors with their affiliations and full address(es) should appear on the title page. On a separate sheet an abstract of no more than 150 words is required. This should succinctly describe the scope of the contribution and highlight significant findings or innovations. It should be written in a style which can easily be translated into French and German.

*The Concise Oxford Dictionary* and Fowler's *Modern English Usage* (both published by Oxford University Press) should be used as the standard for spelling and grammar. Abbreviations should be limited to those generally recognised, or where a frequently occuring term is abbreviated it should, in the first instance, be explained thus "flow injection analysis (FIA) ..." and the abbreviation used thereafter. Abbreviations, for standard measures and units should follow SI recommendations. There are various publications giving guidance on the use of SI units.

References should be indicated in the text by numerals following the author's name, i.e. Skeggs [6]. On a separate sheet of paper, list all references in numerical order thus:

[6] Skeggs, L.T., *American Journal of Clinical Pathology,* 1959, **28**, 311.

Note that journal titles are given in full. Where there is more than one author, the form Foreman et al. should be used in the text but all authors should be named in the list of references. When reference is made to a chapter in a book the reference should take the following form:

[7] Malmstadt, H.V. in "Topics in Automatic Chemistry" Ed. Stockwell P.B. and Foreman J.K. 1978 Horwood, Chichester, pp. 68-70.

Only work which has been published or has been accepted for publication should be cited. Avoid the citation of documents which are subject to restricted circulation, patent literature, unpublished work and personal communications. The latter can be mentioned in the text in parenthesis.

To illustrate a paper line diagrams are preferred to photographs. Photographs should only be used when they significantly add to the discussion. Diagrams, charts and graphs should be carefully drawn in black ink on stout card or heavy quality tracing paper. Most illustrations are reduced for publication; to allow for this originals should be between 16 and 36 cm wide (the depth must not exceed 50 cm). The lettering of diagrams should be sufficiently clear to withstand reduction. Except in the case of proper names, all lettering should be in lower case print. If photographs are used they must be supplied in the form of clear, unmounted, glossy, black and white prints. "Instant" photographs are not normally acceptable. All illustrations must be identified on the reverse showing the figure number and the author's name.

Each illustration should have a fully explanitory caption. Captions should be typed together on a separate sheet of paper; they must *not* be an inseparable part of the illustration.

